# Comparing Continuous with Periodic Vital Sign Scoring for Clinical Deterioration Using a Patient Data Model

**DOI:** 10.1007/s10916-023-01954-z

**Published:** 2023-05-08

**Authors:** Roel V. Peelen, Yassin Eddahchouri, Mats Koeneman, René Melis, Harry van Goor, Sebastian J. H. Bredie

**Affiliations:** 1 Department of Internal Medicine, Radboud University Medical Center, Geert Grooteplein 8, 6525 GA Nijmegen, The Netherlands; 2 Department of Surgery, Radboud University Medical Center, Geert Grooteplein 8, 6525 GA Nijmegen, The Netherlands; 3 Health Innovation Lab, Radboud University Medical Center, Geert Grooteplein 8, 6525 GA Nijmegen, The Netherlands; 4 Department of Geriatrics, Radboud University Medical Center, Geert Grooteplein 8, 6525 GA Nijmegen, The Netherlands

**Keywords:** Continuous monitoring, Clinical deterioration, Track and trigger protocols, Early warning scores, Alarm fatigue

## Abstract

To evaluate a minute-by-minute monitoring algorithm against a periodic early warning score (EWS) in detecting clinical deterioration and workload. Periodic EWSs suffer from large measurement intervals, causing late detection of deterioration. This might be prevented by continuous vital sign monitoring with a real-time algorithm such as the Visensia Safety Index (VSI). This prospective comparative data modeling cohort study (NCT04189653) compares continuous algorithmic alerts against periodic EWS in continuous monitored medical and surgical inpatients. We evaluated sensitivity, frequency, number of warnings needed to evaluate (NNE) and time of initial alert till escalation of care (EOC): Rapid Response Team activation, unplanned ICU admission, emergency surgery, or death. Also, the percentage of VSI alerting minutes was compared between patients with or without EOC. In 1529 admissions continuous VSI warned for 55% of EOC (95% CI: 45-64%) versus 51% (95% CI: 41-61%) by periodic EWS. NNE for VSI was 152 alerts per detected EOC (95% CI: 114-190) compared to 21 (95% CI: 17-28). It generated 0.99 warnings per day per patient compared to 0.13. Time from detection score till escalation was 8.3 hours (IQR: 2.6-24.8) with VSI versus 5.2 (IQR: 2.7-12.3) hours with EWS (P=0.074). The percentage of warning VSI minutes was higher in patients with EOC than in stable patients (2.36% vs 0.81%, P<0.001). Although sensitivity of detection was not significantly improved continuous vital sign monitoring shows potential for earlier alerts for deterioration compared to periodic EWS. A higher percentage of alerting minutes may indicate risk for deterioration.

**Supplementary Information:**

The online version contains supplementary material available at 10.1007/s10916-023-01954-z.

## Introduction

Inpatients’ clinical deterioration is strongly related to morbidity and mortality, and frequently preceded by vital signs abnormalities [1–8]. The last decades, track and trigger protocols based on periodic vital sign measurements have been implemented. These claim to predict in-hospital mortality up to 24 hours in advance [9]. Although such Early Warning Scores (EWS) protocols and afferent rapid response teams (RRT) have been implemented widespread, the evidence regarding their effectiveness is inconclusive [10, 11].

Most EWS protocols limit the assessments to once every eight hours, resulting in hours without observations and possibly failure to timely identify clinical deterioration [9, 12–14]. Even full compliance with such EWS protocols generally provides only last-moment warnings. Moreover, the follow-up in the case of elevated EWS demands extra measurements, which is difficult to adhere to in daily practice [15].

Continuous vital sign monitoring is emerging on the wards with the introduction of wireless monitoring solutions [16–18]. Eliminating the gaps between measurements may help to monitor trends and warn for deterioration at an earlier stage. This would allow clinicians to intervene earlier for better outcomes [19, 20]. A recent review shows evidence for reduced mortality and morbidity after the implementation of continuous monitoring [21]. Other reviewers do warn for the large amount of measurement data, which are difficult to interpret, and which leads to many false positives [22].

Moreover, most EWSs are validated only for periodic vital sign measurements. Few scores are validated in combination with continuous vital sign monitoring which would require an automated real-time algorithm. The Visensia Safety Index (VSI) (OBS Medical, Gloucestershire, UK) is such an algorithm. The VSI works by comparing the combined patterns of continuously monitored vital signs to a database of normal vital signs [23]. The VSI needs a minimum of three vital signs to calculate a score and begin minute-by-minute trending.

In 2018, a continuous vital sign monitoring system with the ViSi Mobile (Sotera Wireless Inc, San Diego, USA) wearable device was introduced at our medical and surgical nurse wards [16]. With this introduction, ICU-admissions were reduced by 36% [24]. This result was obtained with the periodic EWS protocol still in use for assessing the continuous measurements, while provided with trend information. In preparation for the implementation of predictive algorithms for clinical decision-making, we wanted to investigate the added value and influence on workload of continuous vital sign monitoring in combination with an alerting score appropriate for continuous data.

The primary objective of this study is to determine the potential added value of every-minute assessment using an alerting score developed for use with continuous monitoring, in detecting clinical deterioration requiring escalation of care (EOC). We hypothesize that continuous monitoring plus VSI outperforms periodic EWS with continuous vital signs available when it comes to early warning for EOC. We also compare the total number of warnings arising from both scenarios, to explore the potential added workload. As a secondary objective, we explore the potential value of the number of alerting minutes of continuous VSI between patients that require EOC or not.

## Methods

Since June 2018, all patients admitted to two selected wards are routinely connected to the ViSi Mobile device, that continuously measures heart rate, respiration rate, blood oxygen saturation, skin temperature, and cuffless blood pressure [16, 24, 25]. Real-time vitals and waveforms are shown on monitors and every minute a set of vital signs is transferred to a research database. Periodically a set of vital signs of these continuous vital sign measurements is validated by nurses, after which it is automatically registered in the to the Electronic Medical Record (EMR; Epic, Epic Systems Corporation, Verona, Wi, USA), and an EWS is calculated (Modified EWS algorithm, Appendix 1). This study was approved by the Radboud University Institutional Human Research Ethics Committee (METC 2018–4330, NCT04189653). The need for a written consent was waived as the replacement of manual vital sign measurements by automated continuous monitoring was considered the standard of care for these wards.

### Design

This study was performed following the STROBE guidelines for observational cohort studies. With this prospective comparative data modeling cohort study, we explored how often and when warnings were generated on either regular periodic EWS or continuous VSI calculation in well-defined clinical situations requiring EOC. We compared sensitivity, number needed to evaluate (NNE), number of warning scores per patient per day, and the time between initial warning and EOC. Also, the percentage of VSI alerting minutes was compared between patients with or without EOC.

### Participants & clinical data

All patients, 18 years and older, admitted to the 34-bed medical ward or 26-bed gastrointestinal surgical ward of our academic hospital in the Netherlands from August 2018 to July 2019 were included. If patients were not connected within the first 12 hours or had an admission that was estimated to be shorter than 24 hours, they were excluded from the analysis. Relevant changes in clinical condition, interventions, occurrence of complications, new diagnoses and clinical endpoints such as RRT calls, ICU admission, discharge, or death were prospectively recorded in a pseudonymized research database (Castor EDC, Ciwit BV, Amsterdam, NLD) by two MD’s using a purpose-designed electronic form, analyzing all EMR orders and notes.

### Definitions of clinical deterioration

In order to evaluate the continuous monitoring plus VSI, we used the minute-by-minute data to calculate a Visensia Safety Index. This is a commercially available FDA-approved risk score with a CE mark for minute-by-minute assessment of the continuous vital sign monitoring data. The VSI used four vital signs, namely, blood oxygen saturation, respiratory rate, heart rate and blood pressure, but only requires at least three of the four vitals for its calculation. It can also use core temperature as a fifth parameter; however, we did not use this function as this was not continuously measured. The algorithm combines available vitals and constantly scores these against a range of normal vital signs. It also has smooths out extreme measurements to filter for faulty measurements using surrounding reassuring data. A VSI ≥ 3 indicates predicted critical instability within the coming 6 hours [23]. The current protocol for assessment of the clinical status in our hospital is based on periodic calculation of a modified EWS, which also incorporates oxygen delivery and level of consciousness (Appendix 1). For this study the onset of clinical deterioration was defined as an EWS ≥ 6 in the case of usual periodic monitoring, or a VSI ≥ 3 in the scenario with continuous vital sign monitoring, which corresponds in degree of deviation and are for both scores the official point of alarming.

In our modeling, an alerting value of one of these scores starts a ‘warning period’. This warning period can either result in an EOC endpoint, or return to a normal clinical situation without an EOC occurring. A warning period arbitrarily ends after 4 hours. This 4-hour period was chosen as it corresponded to the measurement interval advised by the current protocol for an EWS above 3. If an additional alert (increased EWS or VSI) occurred within that 4-hour period, we considered this alert related to the initial alert. In these cases, the period was lengthened with another 4 hours from the latest alert. To allow comparison, the intervals with continuous monitoring in which no alert occurred were limited to a maximum of 8 hours, which equals the duration of a regular periodic monitoring interval in stable patients.

### Outcome parameters

Possible clinical outcomes were either discharge to home, or a composite endpoint Escalation of Care, defined as either RRT activation, unplanned ICU admission, need for emergency surgery, or death. These events were extracted from the EMR using RRT orders, transfer orders to the CCU or ICU if these were not expected to occur at admission, or surgery orders that were not planned 12 hours ahead of the procedure. When a EOC has occurred, this patient would not be used for further analysis of new EOC’s, with exception of RRT-activations, which were assessed as new escalations if at least 24 hours apart. Detection of EOC was the primary outcome of the study.

### Analysis

Descriptive statistics are presented as the mean with standard deviation (SD) or median with interquartile range (IQR). For confidence intervals of proportions, we used the standard error to calculate a 95% confidence interval (95% CI). For non-normally distributed data, Mann-Whitney U tests were used to detect differences in shape and spread as well differences in medians.

For all warning periods, it was determined whether the elevated EWS or VSI was a true positive, a false positive or true negative warning with respect to a reached EOC. If an EOC occurred outside a warning period, it was counted as a false negative warning. The elapsed time between the initial alert and a successive EOC (true positives) was compared between both scenarios.

As a measure of added workload as result of implemented continuous monitoring, the number needed to evaluate (NNE) were determined as ratio of false to true warnings. Crosstabs were created for warning periods versus EOC to calculate sensitivity or recall (TP/EOC), positive predictive value or precision (PPV = TP/(TP+FP)) and the number of warning periods needed to evaluate (NNE = 1/PPV) to indicate one true warning. To convert this to a scalable clinical number, we normalized the NNE with respect to the number of patient admission days, resulting in a number of warnings per patient per day.

Secondary, using the continuous monitoring data, we divided number of warning minutes by the number of available monitored minutes (fraction of warning minutes) and compared this between patients who ended in EOC or with discharge. This was done for the first 120 admission hours (median admission time) to avoid outliers. In addition, we calculated the fraction of warning minutes during the last three days before an EOC had occurred e.g., 0-24 hours, 25-48 hours and 49-72 hours. This was visualized in Fig. 2 to show the distribution and nature of the data of this new variable.

All statistical analyses were performed using IBM SPSS 23 (SPSS, Inc, Chicago, IL, USA). Figures were made using IBM SPSS 23 and GraphPad Prism 9 (GraphPad Software, San Diego, CA, USA).

## Results

A total of 1529 of 2303 admissions were eligible for analyses because they were connected to continuous monitoring within the first 12 hours of the admission at the ward that lasted for longer than 24 hours. Of these patients, 106 (7%) encountered an EOC. Table 1 shows the descriptive characteristics of the enrolled patients.

**Table 1:** Patient characteristics

**Patient Characteristics**	
**Admissions**	1529
Age (SD)	60.1 (+/- 15.7)
Male (% of total)	774 (51%)
BMI (SD)	26.4 (+/- 4.3)
Internal/Surgery	1031/537
ASA-classification on admission	I: 49 II: 710 III: 785 IV: 24
**Acuity of admission**	
*Planned admission*	*312*
*Emergency admission*	*772*
*Post-surgery or ICU*	*484*
**Patients with monitoring discontinued**	170 (11%)
**Escalations of care**	106 (7%)

Patient characteristics of the eligible inpatients

*BMI* body mass index, *ASA* American Society of Anesthesiology

A total of 38,547 periodic vital sign measurements and 10,074,490 minutes of continuous vital sign monitoring were analyzed across 8,890 patient days. Of the expected continuous vital sign monitoring data (one registration every minute) 79% was available. Common reasons for missing continuous data were unavoidable monitoring interruptions during diagnostics or therapies, the device not being connected to the network, or other technical errors. In 170 patients monitoring was discontinued due to various reasons such as delirium, contact allergies, at the initiation of palliative care, or late opting out of the monitoring. Hereby these patients were also stopped being followed-up. For these patients no further endpoint was registered.

Table 2 depicts the primary outcomes. It shows that sensitivity to detect an EOC was 55% (95% CI: 45-64%) for continuous vital sign monitoring compared to 51% (95% CI: 41-61%) for periodic vital sign measurements. The specificity was 74% (95% CI: 73-74%) for continuous vital sign monitoring and 96% (95% CI: 96-96%) for periodic vital sign measurements. The positive predictive value for an EOC with continuous vital sign monitoring was 0.007 (95% CI 0.005-0.009), versus 0.048 (95% CI 0.035-0.060) with periodic vital sign measurements. With continuous vital sign monitoring, the number of warnings that needed to be evaluated for each EOC was 152 (95% CI: 114-190) compared to 21 (95% CI: 17-28) for periodic vital sign measurements. This translates to 0.99 warnings per patient admission day with continuous vital sign monitoring to evaluate and 0.13 for periodic vital sign measurements.

**Table 2:** Primary outcomes: Periodic EWS vs Continuous VSI for detecting Escalation of Care

**Primary outcomes: Periodic EWS vs Continuous VSI for detecting Escalation of Care**					
Periodic EWS	EOC +	EOC -	Total	Sensitivity	51% (95% CI 41-60%)
Warning	54	1072	1126	Specificity	96% (95% CI 96-96%)
Not warned	52	27344	27396	PPV	0.048 (95% CI 0.035-0.060)
Total	106	28416	28522	NNE	21 (95% CI 17-28)
Continuous VSI	EOC +	EOC -	Total	Sensitivity	55% (95% CI 45-64%)
Warning	58	8726	8784	Specificity	74% (95% CI 73-74%)
Not warned	48	24772	24820	PPV	0.007 (95% CI 0.005-0.009)
Total	106	33498	33604	NNE	151 (95% CI 114-190)

*Primary outcomes* Periodic EWS vs Continuous VSI for detecting Escalation of Care. Relationship between warning periods and Escalation of Care for both periodic EWS and continuous vital sign monitoring in combination with VSI

*EWS* Early Warning Score, *EOC* Escalation of care, *VSI* Visensia safety index, *PPV* Positive Predictive Value or Precision, *NNE* Numbers Needed to Evaluate

**Fig. 1:**
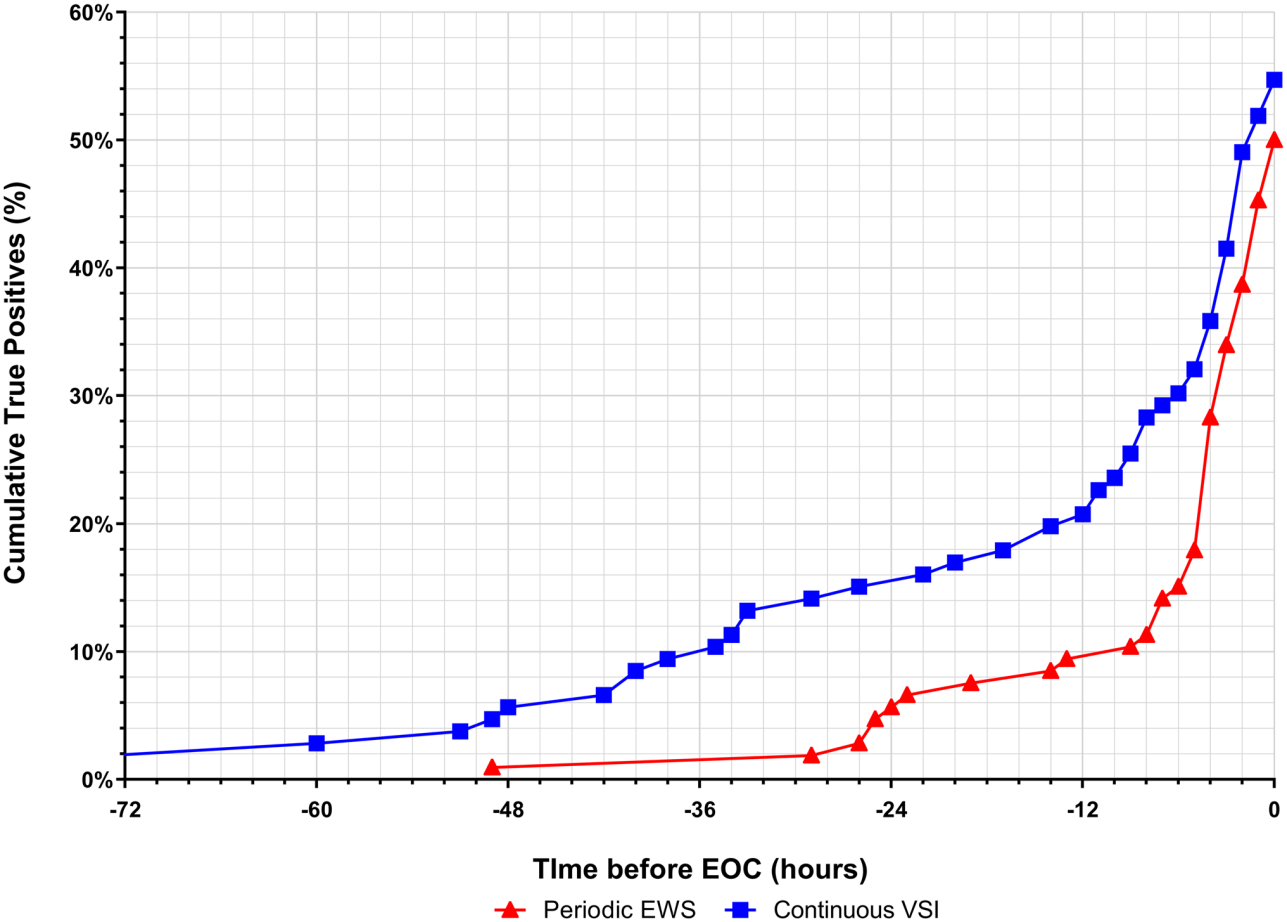
Cumulative true EOC events alerted for by either periodic vital sign measurements with EWS (red line) or continuous vital sign monitoring with VSI (blue line). EWS: Early Warning Score; EOC: escalation of care; VSI: Visensia safety index. X-axis: hours before occurrence of an EOC. Y-axis: cumulative percentage of true detected EOC events

Time from initial warning to EOC is compared in Fig. 1. With both scenarios, approximately half of all EOC could be detected. From 48 hours prior to the endpoint, the total number of EOC detected with continuous vital sign monitoring was larger than that with periodic vital sign measurements (Fig. 1). Continuous vital sign monitoring warned earlier for an upcoming EOC: 8.3 hours (IQR: 2.6-24.8) versus 5.2 hours (IQR: 2.7-12.3) respectively, although this was not statistically significant (P=0.074). A description per different form of EOC can be found in Appendix 2.

**Fig. 2:**
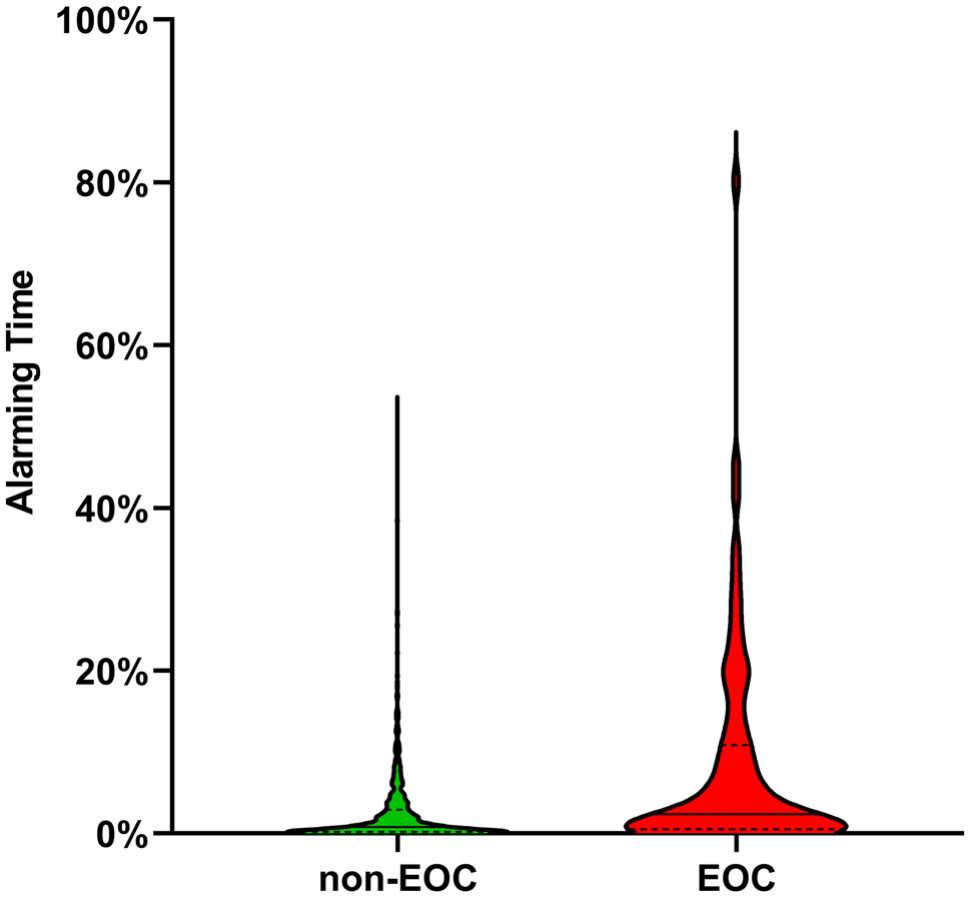
Distribution of the percentage of alarming time in patients with or without EOC. The percentage of alarming time was calculated dividing the alarming minutes by the available monitoring minutes of the first 120 hours of each admission. Non-EOC vs EOC medians are 0.81% (IQR: 0.20-2.92%) vs. 2.36% (IQR: 0.52-10.87%) (P>0.001). EOC: escalation of care, Y-axis: Median percentage of alerting minutes visualized with violin plots, dotted lines inside of the colored area are IQR ranges, solid line is the median

**Fig. 3:**
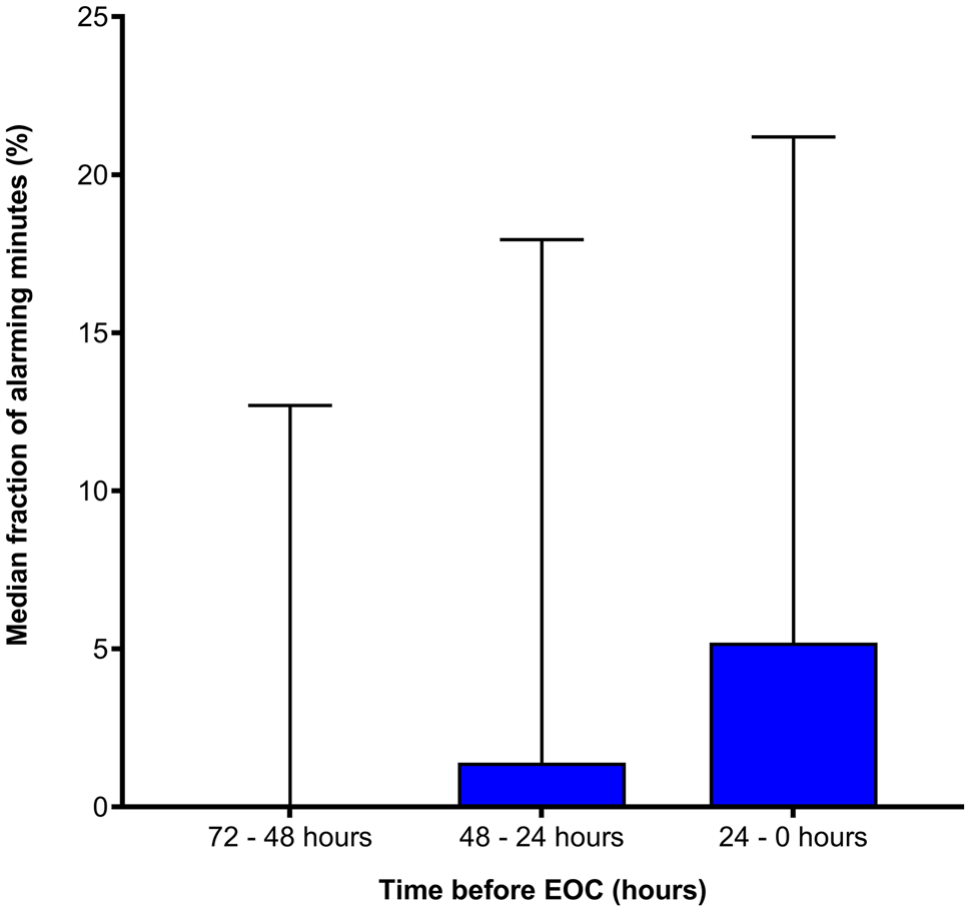
Median of percentages of warning minutes. The percentage was calculated by dividing the warning minutes by the available monitoring minutes in the last 72 hours before EOC. 72-48 hours: 0.00% (IQR: 0.00-12.70%), 48-24 hours: 1.40% (IQR: 0.00-17.95%) 24-0 hours: 5.20% (IQR: 0.00-21.20%). EOC: escalation of care, X-axis: hours before occurrence of an EOC. Y-axis: Median fraction of alerting minutes visualized with boxplots

For our secondary outcome, we observed using continuous vital sign monitoring plus VSI, that the percentage of the alerting time in patients whose admission ended with an EOC was higher than in the patients who were discharged without EOC, shown by a difference in distribution in Fig. 2 (median percentage of alerting time: 2.36% (IQR: 0.52-10.87%) vs 0.81% (IQR: 0.20-2.92%), Mann-Whitney U-test: U 35302.00, Z -4.211, P>0.001). When comparing the fraction of alerting minutes in the last 72 hours for EOC a rising trend in median alerting time from 0.00% (IQR :0.00-12.70%) to 5.20% (IQR: 0.00-21.20%) is seen as shown in Fig. 3.

## Discussion

In this study we explored the potential value and the influence on the workload of a warning score developed for the use with continuous monitoring over periodic EWS calculation in detection of clinical deterioration ending in escalation of care. It was shown that continuous vital signs monitoring in combination with an appropriate risk score algorithm (Visensia Safety Index, VSI) equaled periodic vital sign measurements in terms of sensitivity and has the potential to detect clinical deterioration earlier. However, the PPV is significantly lower than that of periodic monitoring. Continuous monitoring plus VSI resulted in a higher fraction alerting time in patients with an EOC.

Clinical decision making during our observations was based on the EWS protocol, so it was expected that a retrospective modeled score would reach the same sensitivity for EOC as the periodic EWS. Furthermore, the periodic EWS was supported with clinically available continuous monitoring data. Therefore, the added value of the minute-by-minute VSI for indicating clinical deterioration is assumed underestimated. The earlier detection can be explained by the continuous measurements, alerting for deterioration hours before a planned assessment [15].

The use of the VSI has other benefits. It used only four vitals, that can be derived from current automated monitoring systems. Even in case of temporarily unavailability of one of the vitals, the VSI can be calculated. Using fewer vitals than previous studies, our findings are in line with recent reviews, which shows that continuous monitoring detects deterioration earlier, even without advanced analysis [21, 22].

Screening tests for clinical deterioration need to balance between workload and safety i.e., between an acceptable number of false positives and an adequate sensitivity [27–29]. Continuous alerting generates more alerts as the number of measurements is increased. Not only does this increase the risk of alarm fatigue, it also influences the safety of other patients on the ward, increases workload and risks provider burnout [30]. Therefore, false positive alerts should be reduced as far as possible without reducing sensitivity. However, despite the low PPV, we still found no warnings in about 40% of the patients who ended with an EOC. To increase both PPV and sensitivity, more complex analysis and filtering are needed e.g., time-series, trend or variability analysis. This is investigated in recent publications, where sensitivities between 50 and 90% are reported in studies using complex algorithms on continuous monitoring in retrospective scenarios with varying NNE’s [28, 31]. In our study, we observed a 7-fold increase in alerts when using continuous monitoring with VSI. Yet, when calculated down to the number of alarms per patient per day it is just under 1 per day. This is in line, if not better, compared to a recent review on continuous monitoring reporting 2 to 10 warnings per patient per day [17], but leaves room for further needed reduction.

Current literature compares continuous monitoring to periodic EWS and treats both as a surveillance system for acute deterioration needing immediate escalation [21, 22]. There is little attention for the possibility to prevent deterioration using continuous monitoring instead of to detect deterioration. Abnormal monitoring data might need other, possibly less acute follow up then a warning EWS. A trend in monitoring data could initiate a subtle change in therapy, preventing deterioration with less effort and less precipitance [19–21, 32]. In our previous research we found a reduction in RRT-activation and ICU-admissions after implementing continuous monitoring, suggesting prevented escalation by clinicians [24]. In our current analysis we see an earlier detection for RRT-activation and surgery when using continuous alerting. We suggest a focus on using continuous monitoring not to create continuous alerts, but to use it for gradual risk assessment. By guiding clinicians attention to where needed in an early, this could improve workflow and help reduce workload without the nuisance alarms of a warning system.

The fraction of alerting minutes could be such a risk assessment. The higher fraction of alerting minutes in the EOC patient group combined with the ascending trend towards the moment of EOC, suggests that the increasing number of alarms may be a precursor to a dysregulation. These apparent false positives do seem to lead the healthcare provider to the patients in need. Other studies report frequent and significant periods of hypoxemia, tachycardia and hypotension in ward patients, not found with periodic measurements. It is suggested that these could be early signs of deterioration [23, 26, 33]. The higher fraction of alerting minutes and increase toward EOC in our analysis supports that idea. Future research should investigate these alarms and try to correlate duration, frequency and the trend of alerting minutes to clinical interventions and adverse events. These interventions and events could be an intermediate outcome on their own, being one of the earlier steps in the cascade of deterioration, useful for the detecting preventable deterioration.

### Strengths & limitations

This study is relatively large compared to current literature, being based on the clinical course during 8889 admission days [17, 21, 22]. With more than 1500 surgical and medical admissions it is representative as a mixed hospital population. All patients were treated with standard care, and not in a trial context, minimizing bias. With continuous monitoring data available in between periodic EWS, the contrast with continuously calculated VSI could have been more significant if compared with only periodic EWS. Although the modelling was done retrospectively, protocolized gathering of clinical data was done on a daily basis, allowing for more reliable and exact timestamps than previous retrospective analyses. As our analysis is a retrospective simulation, it is a modeling of a complex care system. For this study we used available, off-the-shelve solutions for reproducibility and for a realistic real-world comparison. Thresholds and protocols should be further investigated for further implementation.

### Recommendations for new monitoring protocols

Early detection on its own is not enough to prevent deterioration. Suitable follow-up protocols need to be formed, actuating a proper response [19, 21, 22]. We suggest that track-and-trigger protocols based on continuous monitoring should not solely be used as a warning for impending EOC, but for earlier identification of deterioration. For this a well-formulated intermediate outcome is needed. This could help guiding towards early engagement of high-acuity teams and best-practice approaches [32]. Protocols should be developed with appropriate, scalable follow up actions depending on the level of alarm, e.g., based on fraction of alerting time. Further studies should focus on both patient safety with an increased sensitivity for adverse events, but also on increasing the PPV to secure workability and cost efficiency, following the quadruple aim principle [34].

## Conclusion

Continuous vital sign monitoring with an appropriate warning score (VSI) showed an equal sensitivity to continuous vital sign monitoring with periodically calculated EWS for escalation of care while alarming more frequent. Continuous VSI has the potential to identify clinical deterioration earlier, while using fewer vital sign parameters. In addition, the fraction of alerting minutes may indicate patients at risk for clinical deterioration.

### Supplementary Information

Below is the link to the electronic supplementary material.

Supplementary file1 (PDF 311 KB)

Supplementary file2 (DOCX 16 KB)

## Data Availability

The participants of this study did not give written consent for their data to be shared publicly, so due to the sensitive nature of the research supporting data is not available.
