# Supplementary material for: Comparing Continuous with Periodic Vital Sign Scoring for Clinical Deterioration Using a Patient Data Model
Source: J Med Syst. 2023 May 8;47(1):60. doi: 10.1007/s10916-023-01954-z (PMC10167173; doi:10.1007/s10916-023-01954-z)
Supplement: Supplementary file 1 — Supplementary file1 (PDF 311 KB) [file 10916_2023_1954_MOESM1_ESM.pdf]

Appendix 1. Modified Early Warning Score algorithm and follow-up protocol.

	Score points	3	2	1	0	1	2	3
A	Oxygen delivery, L/min				None	< 5	≥ 5	
	Oxygen saturation, %	≤ 91	92-93	94-95	≥ 96			
B	Respiratory rate, /min	≤ 8		9-11	12-20		21-24	≥ 25
C	Heart rate, /min	≤ 40		41-50	51-90	91-110	111-130	≥ 131
	Systolic blood pressure, mmHg	≤ 90	91-100	101-110	111-219			≥ 220
D	Consciousness				A		Delirious	V/P/U
E	Core temperature, Celsius	≤ 35.0		35.1-36.0	36.1-38.0	38.1-39.0	≥ 39.1	

MEWS	Protocol action
0-2	Next measurement <8 hours
3-5	Next measurement <4 hours, consultation fellow nurse or ward physician
≥6	Measurements every hour, consider continuous vital sign monitoring. Consultation ward physician within 10 min and/or Rapid Response Team (RRT) consultation
	Be also aware of sepsis.

AVPU-score: A; Alert (patient is awake and follows commands), V; Voice (patient does not respond to verbal stimulus), P; Pain (patient responds only to a painful stimulus), U; Unresponsive
