# Supplementary material for: Comparing Continuous with Periodic Vital Sign Scoring for Clinical Deterioration Using a Patient Data Model
Source: J Med Syst. 2023 May 8;47(1):60. doi: 10.1007/s10916-023-01954-z (PMC10167173; doi:10.1007/s10916-023-01954-z)
Supplement: Supplementary file 2 — Supplementary file2 (DOCX 16 KB) [file 10916_2023_1954_MOESM2_ESM.docx]

**Appendix 2**

| Type of Escalation of Care detected | | | | | |
| --- | --- | --- | --- | --- | --- |
|  | **Periodic EWS** | | **Continuous VSI** | | **Total** |
|  | *cases* | *hours* | *cases* | *hours* |  |
| **RRT-activation** | 23 | 6.2 (4.3-13.3) | 24 | 11.0 (4.8-33.6) | 53 |
| **Unplanned ICU-transfer** | 23 | 6.1 (4.5-13.5) | 25 | 5.8 (2.7-25.2) | 31 |
| **Emergency Surgery** | 7 | 3.1 (3.1-12.9) | 7 | 11.9 (8.0-18.6) | 20 |
| **Death** | 1 | - | 2 | - | 2 |

Appendix 2; Table depicting the detected escalation in to the different types of care escalations: Rapid Response Team activation, unplanned ICU transfer, emergency surgery and death. From left to right, the number of detections and the median time from detection until escalation (IQR) are listed for each score, followed by the total number of registered escalations. Median alarming time for death was not calculated due to the limited occurrences.
